# The GRAS gene family in watermelons: identification, characterization and expression analysis of different tissues and root-knot nematode infestations

**DOI:** 10.7717/peerj.11526

**Published:** 2021-05-26

**Authors:** Gongbo Lv, Xing Zheng, Yitian Duan, Yunyong Wen, Bin Zeng, Mingqiang Ai, Bin He

**Affiliations:** 1 College of Life Sciences, Jiangxi Science & Technology Normal University, Jiangxi Key Laboratory of Bioprocess Engineering and Co-Innovation Center for In-Vitro Diagnostic Reagents and Devices of Jiangxi Province, Nanchang, Jiangxi, China; 2 Renmin University of China, School of Information, Beijing, China; 3 Shenzhen Technology University, College of Pharmacy, Shenzhen, Guangdong, China

**Keywords:** Watermelon (*Citrullus lanatus*), GRAS transcription factor, Genome-wide analysis, Root-knot nematode, Expression pattern

## Abstract

The family of GRAS plant-specific transcription factor plays diverse roles in numerous biological processes. Despite the identification and characterization of GRAS genes family in dozens of plant species, until now, GRAS members in watermelon (*Citrullus lanatus*) have not been investigated comprehensively. In this study, using bioinformatic analysis, we identified 37 GRAS genes in the watermelon genome (ClGRAS). These genes are classified into 10 distinct subfamilies based on previous research, and unevenly distributed on 11 chromosomes. Furthermore, a complete analysis was conducted to characterize conserved motifs and gene structures, which revealed the members within same subfamily that have analogous conserved gene structure and motif composition. Additionally, the expression pattern of ClGRAS genes was characterized in fruit flesh and rind tissues during watermelon fruit development and under red light (RL) as well as root knot nematode infestation. Finally, for verification of the availability of public transcriptome data, we also evaluated the expression levels of randomly selected four ClGRAS genes under RL and nematode infection by using qRT-PCR. The qRT-PCR results indicated that several ClGRAS genes were differentially expressed, implying their vital role in RL induction of watermelon resistance against root-knot nematodes. The results obtained in this study could be useful in improving the quality of watermelon.

## Introduction

Transcription factors (TF) are key proteins that recognize and bind to an enhancer or promotor sequences in the typical upstream of the target gene and precisely stimulate or inhibit the transcription of the genes, protein synthesis, and subsequently alter cellular function. TF plays a crucial role in plant growth, primarily participate in the development and physiological processes. Since the first TF was found in *Zea mays*, plenty of TF has been proven to possess various functions in higher plants growth and development, such as WRKY, bZIP, AP2/ERF, MADS, SBP, and GRAS (Alves et al., 2013; Javier Paz-Ares et al., 1987; Pandey & Somssich, 2009; Theissen, Melzer & Rumpler, 2016). The GRAS gene family is a critical plant-specific gene family of putative TF, whose name derives from the acronym for the first isolated three members: GAI (gibberellic acid insensitive), RGA (repressor of GA1-3 mutant), and SCR (scarecrow) (Bolle, 2004). Generally, GRAS proteins harbor 400–770 amino acid residues and exhibit considerable properties at the variable N-terminal and highly conserved C-terminal. There are five pivotal domains in the conserved C-terminal of GRAS proteins with the following order: LHR I (leucine heptad repeat I), VHIID, LHR II (leucine heptad repeat II), PFYRE and SAW (Bolle, 2004). Among them, VHIID is flanked by LHRI and LHRII, and is proven to be vital for protein–protein interactions (Pysh et al., 1999); LHR is shown to have a significant role in protein homodimerization (Tian et al., 2004). Owing to its highly variable nature of length and sequence, the N-terminal region is a key part of the GRAS protein to perform specific functions (Tian et al., 2004). It also interacts with various target proteins to work together to present protein specificity and play an essential role in signaling pathways (Hirano et al., 2017; Sun, Jones & Rikkerink, 2012).

Up to now, the GRAS family has at least 10 established subfamilies, DELLA, LISCL, SCR, HAM, PAT1, SCL4/7, DLT, SHR, Os4, and SCL3, most genes within these families were functionally characterized (Fu et al., 2002; Morohashi et al., 2003; Stuurman, Jaggi & Kuhlemeier, 2002; Tong et al., 2009; Torres-Galea et al., 2006). For instance, it has been reported that LlSCL modulates the premeiotic phase of anthers, and is related to transcriptional regulation during the microsporogenesis of lily anther (Morohashi et al., 2003). Several genes like GAI, RGA, and RGL of the DELLA subfamily were demonstrated as repressors of gibberellin signaling in the growth of *Arabidopsis thaliana* (*A. thaliana*) (Hirsch & Oldroyd, 2009). Gain- or loss-of-function mutants of the DELLA genes in maize, wheat, and barley cause GA-insensitive/-nonresponsive dwarf or GA constitutive response phenotypes (Chandler et al., 2002; Peng et al., 1999; Winkler & Freeling, 1994). There was also a report regarding the DELLA members can mediate jasmonate signaling by competitive binding to jasmonate ZIM-domain protein (Hou et al., 2010). Moreover, HAM is confirmed to be involved in the proliferation of meristematic cells, chlorophyll synthesis and polar organization (Bolle, 2004; Schulze et al., 2010). Another member of the GRAS family, DLT, plays positive roles in phytohormone (brassinosteroid) signaling in rice (Tong et al., 2009).

With the deep exploration of GRAS transcription factors in past decades, the unique functions of GRAS proteins in various physiological processes and regulatory networks of the plant have become apparent. Distinct functions of it have been emphasized in regulating plant development and signal transduction, such as stem cell maintenance, phytochrome A signal transduction, radial root patterning, axillary shoot meristem formation, meristem maintenance, phytohormones (gibberellins) signal transduction, light signaling, plant disease resistance, and abiotic stress responses microspore as well as male gametogenesis (Bolle, 2004; Morohashi et al., 2003; Stuurman, Jaggi & Kuhlemeier, 2002; Torres-Galea, Hirtreiter & Bolle, 2013). Specifically, PAT1 and SCL21 are positive regulators of phyA-signaling pathway (Cordelia Bolle & Chua, 2000; Torres-Galea, Hirtreiter & Bolle, 2013). Two GRAS proteins, SCR and SHR are found to be associated with the control of root radial patterning and root growth in *A. thaliana* via forming the SCR/SHR complex (Hirsch & Oldroyd, 2009). Besides, SCL14 protein participation in the activation of a broad-spectrum detoxification network by interacting with TGA transcription factors in *Arabidopsis* (Fode et al., 2008); the GRAS protein RAD1 was reported to interact with the GRAS transcription factors, RAM1 and LjNSP2, respectively, thus it is beneficial for the existence of a GRAS protein-protein interaction network in arbuscule development (Xue et al., 2015). Meanwhile, more than a dozen GRAS gene families in various plants have been identified in the past 5 years. A total of 12 GRAS genes identified from *Tamarix hispida*, 46 GRAS genes from orchardgrass, 150 GRAS genes from *Gossypium hirsutum L*, 87 putative GRAS genes in the *Brassica napus*, 23 GRAS genes in *Chrysanthemum morifolium*, and 52 GRASs were identified in potato and walnut (Gao et al., 2018; Guo et al., 2019; Quan et al., 2019; Wang et al., 2018; Wang et al., 2019; Xu et al., 2020; Zhang et al., 2018). Moreover, *Chinese cabbage*, *Grapevine*, and *Zea mays L* have also been investigated; however, research on the evolution, classification, and functional characteristics of the GRAS family in watermelon is scarce (Grimplet et al., 2016; Guo et al., 2017; Song et al., 2014).

Watermelon is an economically high valuable cucurbit crop produced worldwide. During the growing and developmental processes, watermelon is susceptible to different abiotic and biotic stress, like the root-knot nematodes (RKNs, *Meloidogyne incognita*). *Meloidogyne incognita*, one of the sedentary endoparasitic nematodes, is the most devastating plant pathogen, which might improve susceptibility to other pathogenic diseases and could lead to a decline in the crop yield (Bebber, Holmes & Gurr, 2014; Yang et al., 2019). For example, nematodes can exacerbate the deleterious effects of pathogenic bacteria and soil-inhabiting fungal species, and thus damage root systems (Jones et al., 2013). Despite this, the information about the potential molecular mechanism of ClGRAS genes in watermelon under root-knot nematode infestation is still limited. Herein, we perform a genome‑wide identification and characterization of the GRAS gene family in watermelon by using bioinformatics methods, composing a systematic analysis and identification of phylogenetic relationships, protein and gene structures, GO annotation, and tissue expression patterns. Furthermore, we also determined the expression levels of watermelon ClGRAS genes under RKN infection by using the RNA-Seq data, in an attempt to understand their possible roles in biotic stress.

## Materials & Methods

### Identification, characterization, and chromosomal location of watermelon ClGRAS genes

The newest versions of the genome annotations of watermelon were downloaded from the Cucurbit Genomics Database (http://www.cucurbitgenomics.org/). The latest HMM (Hidden Markov Model) model downloaded from the Pfam database (http://pfam.xfam.org/) was employed to search members of the GRAS transcription factor with a cut-off E-value of 1e^−10^ with GRAS domain (PFD03514) (El-Gebali et al., 2019). The GRAS protein sequences of *Arabidopsis* were obtained from the Arabidopsis Information Resource (https://www.arabidopsis.org/index.jsp) and rice GRAS sequences were obtained from Rice Gene Annotation Project (http://rice.plantbiology.msu.edu/index.shtml), and both of them were used as queries to explore the Cucurbit Genomics database by the default parameters. Combining with the utilization of the Pfam database and the Conserved Domain Database (CDD, https://www.ncbi.nlm.nih.gov/Structure/cdd/wrpsb.cgi), only those sequences having a full-length GRAS domain were chosen as ClGRAS proteins and applied to the subsequent analyses.

The prediction of the physical and chemical features of ClGRAS proteins, such as molecular weight (MW) and the theoretical isoelectric point (PI) values were calculated by ExPASy (https://web.expasy.org/protparam/). To verify the subcellular localization of the identified ClGRAS proteins, LocTree3 Prediction system (https://rostlab.org/services/loctree3/) and WoLF PSORT Prediction (https://wolfpsort.hgc.jp/) were applied to predict the protein sequences. To identify the chromosomal locations of all ClGRAS genes in watermelon, the information of locus coordinates was obtained from the genomic sequences. MapChart software was used for the drafting of ClGRAS genes’ chromosomal positions and relative distances on the basis of their ascending order of physical position (bp) (Voorrips, 2002).

### Multiple sequence alignment and phylogenetic analysis of GRAS proteins

Multiple sequence alignments of GRAS proteins in watermelon, rice, and *Arabidopsis* were performed using ClustalW (http://www.ebi.ac.uk/Tools/msa/clustalw2/). *Arabidopsis* and rice are generally used model plant species for exploring genetic relationships. MEGA X was further applied to establish an unrooted Maximum Likelihood (ML) phylogenetic tree with a pairwise deletion option and Poisson correction model (Kumar et al., 2018). Bootstrap analysis with 1,000 replicates was employed to examine the statistical reliability and choose the best results. The phylogenetic tree was visualized by the FigTree v1.4.3 program. Thereafter, members of watermelon ClGRAS gene families were further divided into diverse subfamilies based on well-established classification in other species (Huang et al., 2015; Liu & Widmer, 2014; Zhang et al., 2018).

### Analysis of conserved motifs and gene structures

To determine the conserved motifs of each GRAS gene in watermelon, deduced GRAS protein sequences were submitted to MEME (version 5.1.1, http://meme-suite.org/tools/meme), with the default parameters except the number of motifs was chosen 6 (Bailey et al., 2009). To elaborate exon-intron organization for each ClGRAS gene, we downloaded the coding sequences (CDSs) and corresponding genomic sequences of ClGRAS genes in watermelon from the Cucurbit Genomics Database. Hereafter, both of them were subjected to the Gene Structure Display Server 2.0 (GSDS 2.0, http://gsds.cbi.pku.edu.cn), which was conducted and displayed by comparing CDSs and their corresponding genomic sequences.

### Gene ontology annotation

With the intention for functional annotation identification of ClGRAS proteins, 37 watermelons ClGRAS proteins sequences were uploaded to Goplot (an R package) and were utilized for Gene Ontology (GO) analysis, and subsequently for mapping and annotation (Walter, Sanchez-Cabo & Ricote, 2015). The GO analysis of 37 watermelon ClGRAS proteins contains three parts such as biological process, molecular function, and cellular component.

### Analysis of the protein–protein interaction network

Protein-protein interaction (PPI) data were acquired from the online database *STRING* (https://string-db.org/cgi/info.pl), an open-source software interface for predicting and visualizing sophisticated networks. The data for PPI was gathered from text mining of literature from peer-reviewed journals, containing the physical interactions and enzymatic reactions found in signal transduction pathways, computational predictions including those based on genomic context analysis as well as derived from analyses of co-expressed genes (Raman, 2010). The PPI data was preprocessed, containing removing redundancy and self-loops. Targets with a high confidence score >0.7 were selected to generate the PPI networks. PPI networks were visualized in Cytoscape software with the nodes representing proteins/genes and the edges indicating interactions between any two proteins/genes.

### Expression analysis of ClGRAS genes during watermelon fruit development and root-knot nematode infection

The transcriptome data of watermelon fruit flesh and rind tissue sampled at 10, 18, 26, and 34 days after pollination (DAP) under BioProject ID SRP012849, were downloaded from the National Center for Biotechnology Information Sequence Read Archive (Bethesda, MD, USA) database. The reference transcriptome was chosen all the annotated gene sequences of the reference genome from watermelon cultivar 97103. In addition, for assessing the expression levels of watermelon ClGRAS genes during RKN infection, the transcriptome data of RKN infection were downloaded from the genome sequence Archive in the BIG Data Center GSA database (https://bigd.big.ac.cn/gsa/browse) under the accession numbers of CRA001311 and CRA001312. It was composed of four different treatments, CK (white light and water solution), RL (red light treatment and water solution), RKN (white light and *M. incognita* infection), and RL+RKN (RR, red light treatment, and root-knot nematode *M. incognita* infestation). Gene expression levels were estimated and normalized with TopHat/Cufflinks pipeline with FPKM (Fragments Per Kilobase of transcript per Million mapped reads) values extracted from the above-mentioned transcriptome data (He et al., 2015). The log2-transformed FPKM values were employed to draw a heatmap to depict the expression of each ClGRAS gene by using the RStudio (version 1.1.463; RStudio, Boston, MA, USA).

To further comprehensively analyze the expression profiles of ClGRAS genes in watermelon, we performed qRT-PCR experiments of four randomly selected genes (including ClGRAS2, 18, 28, and 33). We extracted total RNA from roots and leaves under four treatments (CK, RKN, RL, and RR, respectively) via using the TRIzol Reagent (Takara, Beijing, China) following the manufacturers’ recommendations. Then, Reverse transcription was performed by PrimeScript RT reagent Kit (TaKaRa, Beijing, China). CFX96 Real-Time PCR Detection System (Bio-Rad, Hercules, CA, USA) was used to perform the qRT-PCR analysis. The watermelon 18S ribosomal RNA (18S rRNA) was chosen as the endogenous control (Dong & Shang, 2013). Specific primers of ClGRAS genes used for qRT-PCR were designed by Primer Premier 6.0 and listed in Table S1. Specifically, qPCR was performed in a 15 μL reaction mixtures containing 7.5 μL 2× SYBR® Premix Ex Taq™ II (TaKaRa, Beijing, China), 1 μL cDNA template, 0.3 μL each gene-specific primer, and 5.9 μL ddH2O. The reaction conditions were 30 s at 94 °C, 45 cycles of 20 s at 94 °C, 20 s at 55 °C, and 30 s at 72 °C. The melting curves were analyzed from 60 °C to 95 °C to observe the specificity of the PCR products. Three biological replicates were conducted for each treatment, and each composed of three technical replicates. Expression levels were determined as the mean across the three replicates and the relative expression between samples was calculated using the comparative 2^−ΔΔCT^ method (Livak & Schmittgen, 2001). The relative expression levels of CK were determined as 1 for comparison.

## Results

### Genome‑wide identification and characterization of GRAS family members in watermelon

Candidate ClGRAS genes were identified from the watermelon genome using the Base Local Alignment Search Tool (BLAST) with the query sequences of *Arabidopsis* and rice GRAS genes (Altschul et al., 1997). Subsequently, the retrieved sequences were submitted to the CDD and Pfam databases to confirm the presence of conserved domains. A total of 37 candidate ClGRAS genes were identified in the watermelon genomes and named according to their chromosomal locations (Table 1). The length of 37 ClGRAS proteins ranged from 226 (ClGRAS32) to 859 (ClGRAS15) amino acid residues.

**Table 1:** The information of GRAS family members identified from *Citrullus lanatus* genome.

Gene name	Gene ID	Map position (bp)	CDS length	PL (aa)	MW (kDa)	pI	SCLpred
ClGRAS1	Cla004946	Chr1:709117:710859:-	1761	586	65	5	chlo
ClGRAS2	Cla003932	Chr1:6496817:6498577:-	1743	580	63.41	6.33	cyto
ClGRAS3	Cla008451	Chr1:9138367:9139512:+	1146	381	43.71	6.01	nucl
ClGRAS4	Cla014014	Chr1:27370177:27371586:-	1410	469	52.59	6.24	chlo
ClGRAS5	Cla014025	Chr1:27482392:27483879:-	1488	495	55.97	5.26	nucl
ClGRAS6	Cla014205	Chr1:29093095:29094897:+	1803	600	66.22	4.5	nucl
ClGRAS7	Cla014396	Chr1:30469652:30471298:+	1647	548	60.97	6.32	nucl
ClGRAS8	Cla007701	Chr2:461095:463237:+	1638	545	60.85	6.55	chlo
ClGRAS9	Cla001409	Chr2:14058035:14059672:+	1314	437	48.99	6.18	nucl
ClGRAS10	Cla020203	Chr2:22341396:22344886:-	2136	711	80.86	5.37	nucl
ClGRAS11	Cla013317	Chr2:30410140:30412275:-	1518	505	57.45	6.11	nucl
ClGRAS12	Cla008228	Chr3:1270977:1273108:-	1416	471	52.96	6.24	nucl
ClGRAS13	Cla019759	Chr3:9824203:9826011:+	2052	683	76.66	5.48	nucl
ClGRAS14	Cla006329	Chr3:22584015:22585430:+	1809	602	65.51	4.87	cyto
ClGRAS15	Cla012151	Chr4:15565656:15568669:+	2580	859	92.89	6.6	nucl
ClGRAS16	Cla013228	Chr5:13204110:13205825:+	1716	571	62.12	5.15	nucl
ClGRAS17	Cla020582	Chr5:28484479:28485885:-	1407	468	52.55	5.37	nucl
ClGRAS18	Cla007022	Chr6:895868:897617:-	2055	684	74.69	6.13	nucl
ClGRAS19	Cla006665	Chr6:2868528:2870582:+	1350	449	50.84	5.27	nucl
ClGRAS20	Cla012302	Chr6:18747371:18749081:-	1608	535	58.23	5.37	nucl
ClGRAS21	Cla019248	Chr6:26370480:26372015:+	1536	511	56.69	6.13	nucl
ClGRAS22	Cla014540	Chr7:21816103:21817704:-	1332	443	48.6	6.15	chlo
ClGRAS23	Cla014537	Chr7:21874598:21875965:-	1662	553	60.7	4.57	cyto
ClGRAS24	Cla014516	Chr7:22268358:22270019:+	1368	455	51.22	6.94	chlo
ClGRAS25	Cla005438	Chr7:28146808:28148139:+	1602	533	59.47	6.59	chlo
ClGRAS26	Cla013954	Chr8:14362701:14364152:-	1452	483	54.17	6.23	cyto
ClGRAS27	Cla015549	Chr9:524467:526282:+	1383	460	51.37	6.11	nucl
ClGRAS28	Cla015143	Chr9:4007318:4009042:+	1713	570	64.06	4.67	nucl
ClGRAS29	Cla015142	Chr9:4010756:4012468:+	1713	570	64.68	4.9	nucl
ClGRAS30	Cla014874	Chr9:7010796:7012178:-	1725	574	65.95	5.21	cyto
ClGRAS31	Cla015025	Chr9:8804397:8806109:+	1557	518	58.11	7.47	nucl
ClGRAS32	Cla005646	Chr10:3004822:3007158:-	801	266	31.08	10.16	nucl
ClGRAS33	Cla004491	Chr10:4205812:4207449:-	1956	651	71.89	5.53	nucl
ClGRAS34	Cla004422	Chr10:4965356:4967311:-	1638	545	60.76	5.81	chlo
ClGRAS35	Cla004408	Chr10:5184892:5186091:+	2337	778	85.55	5.59	nucl
ClGRAS36	Cla017902	Chr10:27128946:27131213:+	2268	755	85.47	5.7	nucl
ClGRAS37	Cla011849	Chr11:2296856:2299856:+	2022	673	74.03	5.1	chlo

**Note:**

PL, Protein length; MW, Molecular weight; pI, Isoelectric point; SCLpred, Predicted subcellular localization; nucl, nucleus; chlo, chloroplast; cyto, cytoplasm.

**Figure 1:**
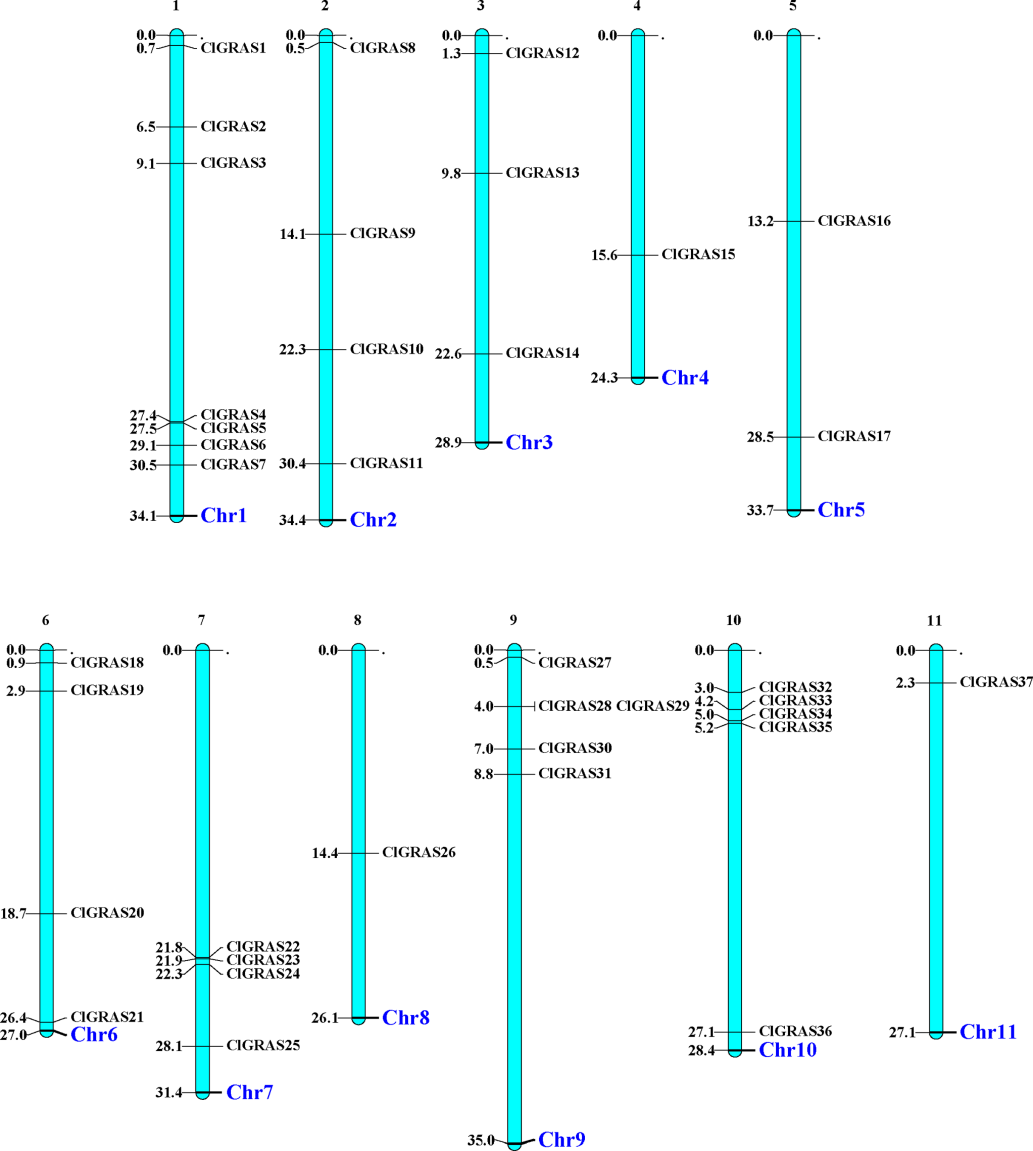
The chromosomal locations of ClGRAS genes in the watermelon genome. Chromosomes 1–11 are depicted as bars. The ClGRAS genes are indicated by black lines.

The distribution of 37 ClGRAS genes on 11 chromosomes was visualized by the MapChart program. It can be seen that all of the 37 ClGRAS genes are unevenly distributed. In addition, most of ClGRAS genes appeared to congregate on the proximate or the distal ends of the chromosomes (Fig. 1). There were 7, 5, and 5 ClGRAS genes mapped onto chromosomes 1,9 and10 respectively, whereas chromosomes 4, 8, and 11 harbored just one ClGRAS gene.

The ExPASy online tool was used to predict the basic physicochemical properties of 37 ClGRAS proteins. As the results showed in Table 1, the molecular weights of these ClGRAS proteins were ranged from 31.08 (ClGRAS32) to 92.89 (ClGRAS15) kDa. Moreover, the majority of the ClGRAS proteins exhibited acidic isoelectric points (less than 6.94), with the lowest being 4.50 (ClGRAS6), only two proteins had alkaline isoelectric points of more than 7.47 (ClGRAS31), of which ClGRAS32 was the highest at 10.16.

Based on the predicted subcellular localization results, ClGRAS proteins were located in the nucleus, chloroplast, and cytoplasm, more than half of it distributed in the nucleus, illustrating that these proteins as transcription factors play a transcriptional regulatory role directly in the nucleus. Detailed information of ClGRAS genes in the watermelon is presented in Table 1.

### Classification and phylogenetic analysis of the GRAS genes

**Figure 2:**
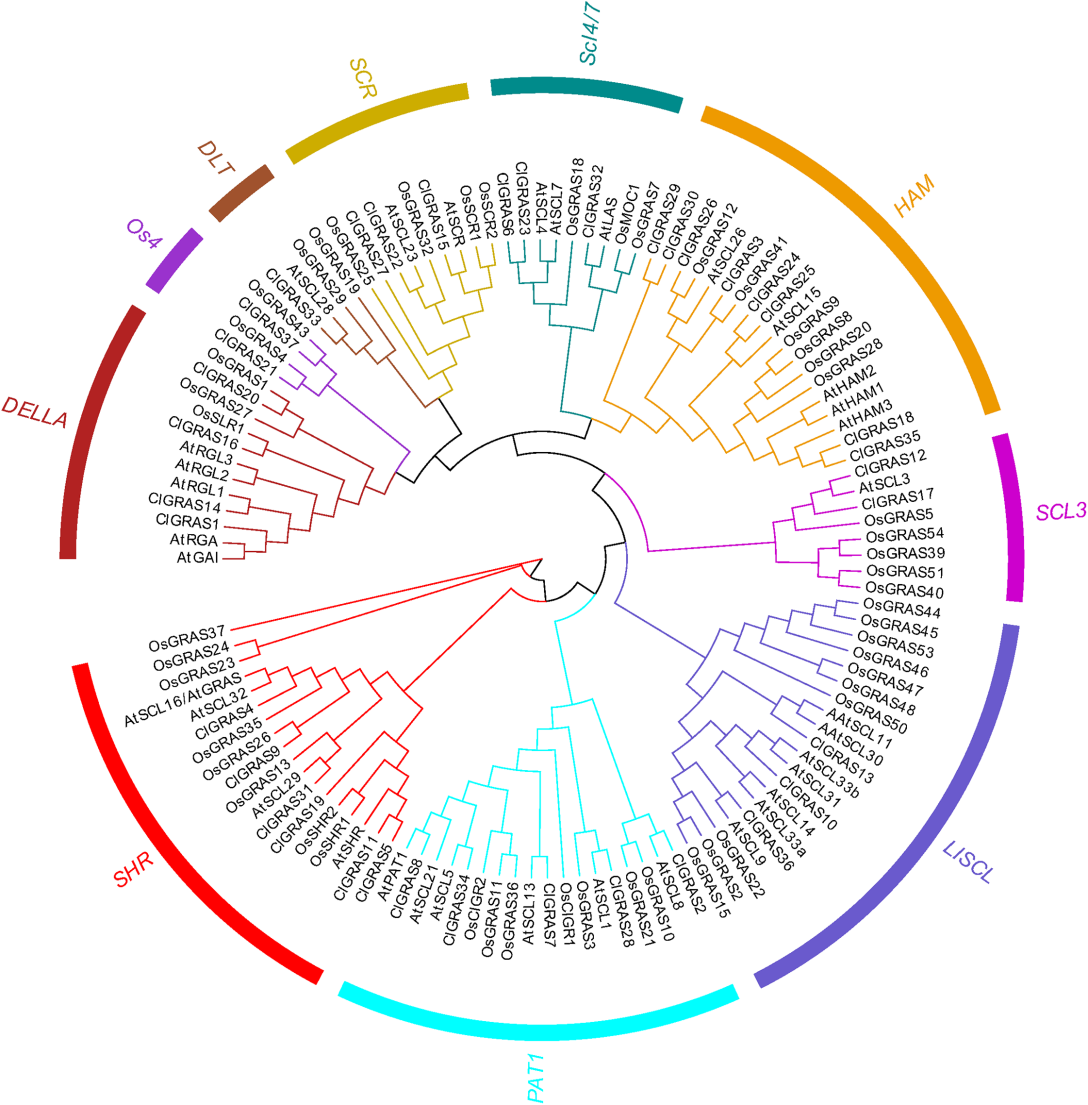
The phylogenetic tree construction of GRAS genes among watermelon, rice and *Arabidopsis*. A Maximum Likelihood (ML) phylogenetic tree of all detected GRAS genes was constructed, using MEGA X program with bootstrap analysis (1,000 replicates). GRAS genes in the phylogenetic tree were clustered into ten distinct subfamilies with different color.

To evaluate the phylogenetic relationships among the ClGRAS gene members, all the genes from watermelon, rice, and *Arabidopsis* were aligned by the Maximum Likelihood method to construct an un-rooted phylogenetic tree. ClGRAS genes in the three species were classified into ten groups as shown in the phylogenetic tree in Fig. 2. Eight watermelon ClGRAS proteins were belonging to the HAM subfamily and constituted the largest subgroup of the phylogenetic analysis, while the DTL subfamily comprised only one member (ClGRAS33). Concerning the HAM subfamily, ClGRAS genes were clustered and homologous with rice and *Arabidopsis* GRAS genes, implying that they could be derived from gene duplications of the same gene locus during the evolution of the watermelon genome. These results indicated that these paralogous genes may play an identical functional role in the growth of watermelon. Apart from this, the un-rooted tree containing 6, 5, 3, 2, 4, 3, 2, and 3 ClGRAS members in the SHR, PAT1, LISCL, SCL3, DELLA, SCR, Os4, and SCL4/7 subcategories, respectively (Fig. 2).

### Motif composition and Exon-intron structures of ClGRAS gene families

**Figure 3:**
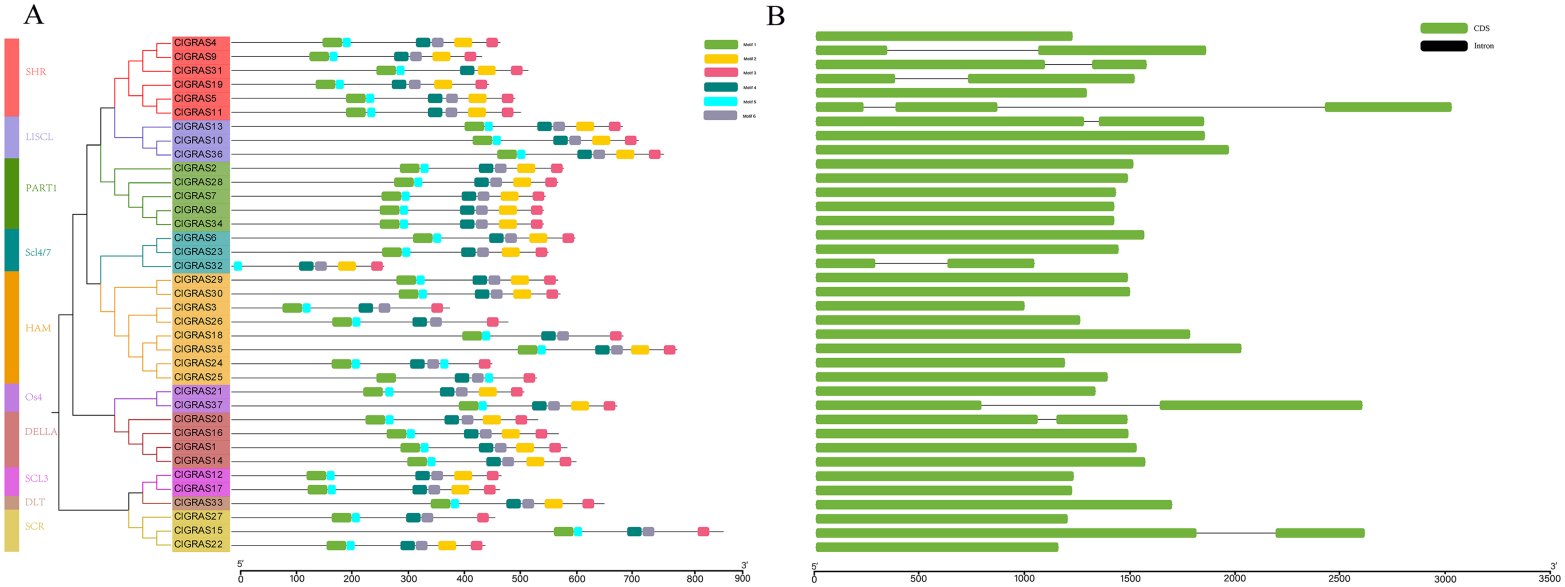
Conserved motifs and Exon-intron structures analysis of the GRAS gene family in watermelon. (A) All conserved motifs of the ClGRAS proteins were identified by the MEME program. Motif compositions: Protein sequences are indicated by thick gray lines, and the conserved motifs are represented by different colored boxes. The length (amino acids) of the protein and motif can be estimated using the scale bar at the bottom. (B) The gene structure analysis of ClGRAS proteins based on their phylogenetic relationships.

A total of six conserved motifs were identified in the ClGRAS proteins and their detailed consensus sequence information is listed in Table 2. According to the phylogenetic tree and conserved motifs (Fig. 3A), we could know that the same group of ClGRAS genes shares similar conserved motifs organization concerning either motif number or gene length, which revealed that there could be similar genetic functions. For instance, ClGRAS3, ClGRAS18, and ClGRAS26, ClGRAS27, and ClGRAS15 have 5 motifs within the same subgroup. There were 4 motifs (motif 2, 3, 4, 6) in the C-terminal region of most ClGRAS proteins, while motif 1 and 5 were found in the N-terminus. Almost all ClGRAS proteins harbored motif 2, 3, 4, and 6 in the C-terminal region, while the motifs across the different proteins were variable in N-terminal. These results are in line with previous research that the C-terminus might be more conserved than the N-terminus via evolution (Fuxreiter, Simon & Bondos, 2011). Moreover, it has previously been demonstrated that variety in C-terminal domains of GRAS proteins can increase the functional versatility and the complexity of biological networks of the GRAS proteins (Sun, Jones & Rikkerink, 2012; Sun et al., 2011). Furthermore, the amino acid frequency at six conserved motifs of ClGRAS proteins was investigated and then downloaded the LOGO of these protein motifs. It indicated that the amino acid frequency of the conserved motifs is not consistent in different ClGRAS proteins (Fig. S1).

**Table 2:** The motifs identified in ClGRAS proteins.

Motif	Conserved amino acid sequences	E-values	Sites	Width
1	PYLKFAHFTANQAILEALEGEDRVHIIDFDIMQGL	3.8e−494	27	35
2	NVVACEGAERVERHETLGKWRARMERAGFKPV	9.4e−280	30	32
3	EENGCLVLGWKERPLVAASAW	4.1e−257	29	21
4	LRLIKSLNPKIVTVVEQEANHNGPFF	1.6e−241	25	26
5	QWPTLIQALALRPGG	1.9e−173	27	15
6	RFVEALHYYSAJFDSLDASLP	2.1e−221	26	21

In order to verify the reliability of the phylogeny analysis and to elucidate the protein functions, as well as structural diversity of ClGRAS genes, gene structures, and conserved motifs in the 37 ClGRAS proteins, were identified using the GSDS 2.0 website and MEME program. As shown in Fig. 3B, the number of introns ranged from zero to 2. A considerable proportion of ClGRAS genes do not contain introns, and an analogous phenomenon of deletion of introns was observed in the genomes of other species. Eight genes such as ClGRAS9, ClGRAS13, ClGRAS15, ClGRAS19, ClGRAS20, ClGRAS31, and ClGRAS37 possess one intron each, whereas just one gene (ClGRAS11) consists of 2 introns. It should be noted that some genes such as ClGRAS13 and ClGRAS31shared similar intron numbers but with different intron lengths. On the whole, ClGRAS members belonging to the identical subfamily on watermelon had similar exon-intron structures.

### Gene ontology annotation

**Figure 4:**
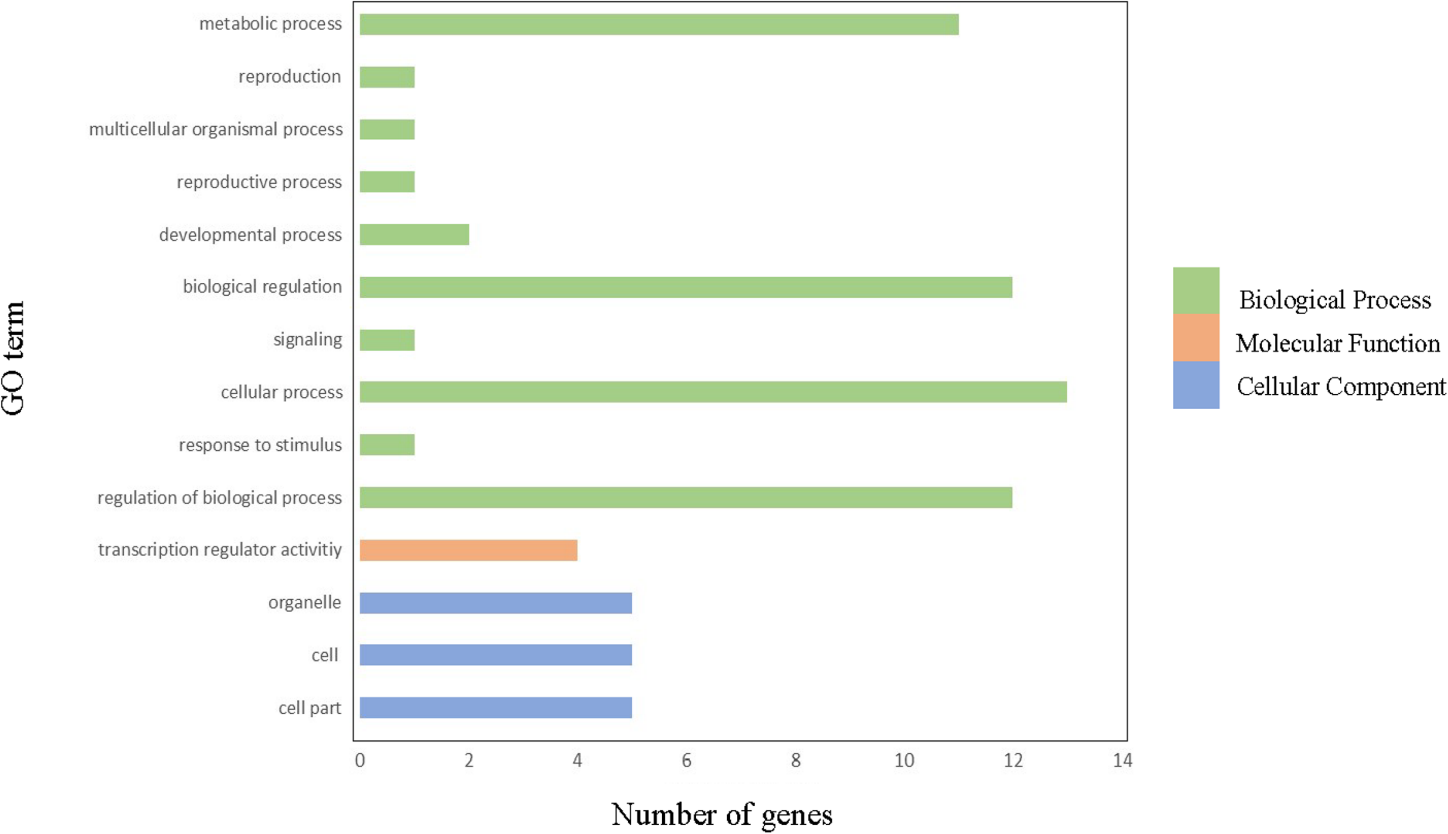
Gene ontology annotations of GRAS proteins in watermelon. There are three parts in this figure: molecular function (MF), cellular component (CC), and biological processes (BP).

In order to gain further insight into which biological processes these ClGRAS genes involved, a GO annotation analysis of the ClGRAS protein was performed and the GO number was present in Table S2. According to the results obtained, ClGRAS proteins were involved in many biological processes (Fig. 4). The many ClGRAS proteins are responsible for cellular processes (GO: 0009987), regulation of biological processes (GO: 0050789), and biological regulations (GO: 0065007). Also over 10 proteins were involved in the metabolic processes (GO: 0008152). It is worth noting that the number of ClGRAS proteins localized in the organelle (GO: 0043226) is the same as that of the cell (GO: 0044464) and cell part (GO: 0005623), which showed that ClGRAS proteins perform a functional role in these locations. Additionally, a moderate number of ClGRAS proteins might be associated with transformation regulatory activity (GO: 0140110). Nonetheless, just a few ClGRAS proteins respond to stimulus (GO: 0050896) and play role in signaling (GO: 0023052) and reproductive processes (GO: 0022414). Thus, according to the above-mentioned analysis of the biological processes of ClGRAS genes, the multi-functional role of watermelon ClGRAS proteins could be involved in various biological processes, such as cellular/metabolic processes, and had functions in processes of signal transduction as well as the stress response.

### Interaction network of ClGRAS proteins in watermelon

**Figure 5:**
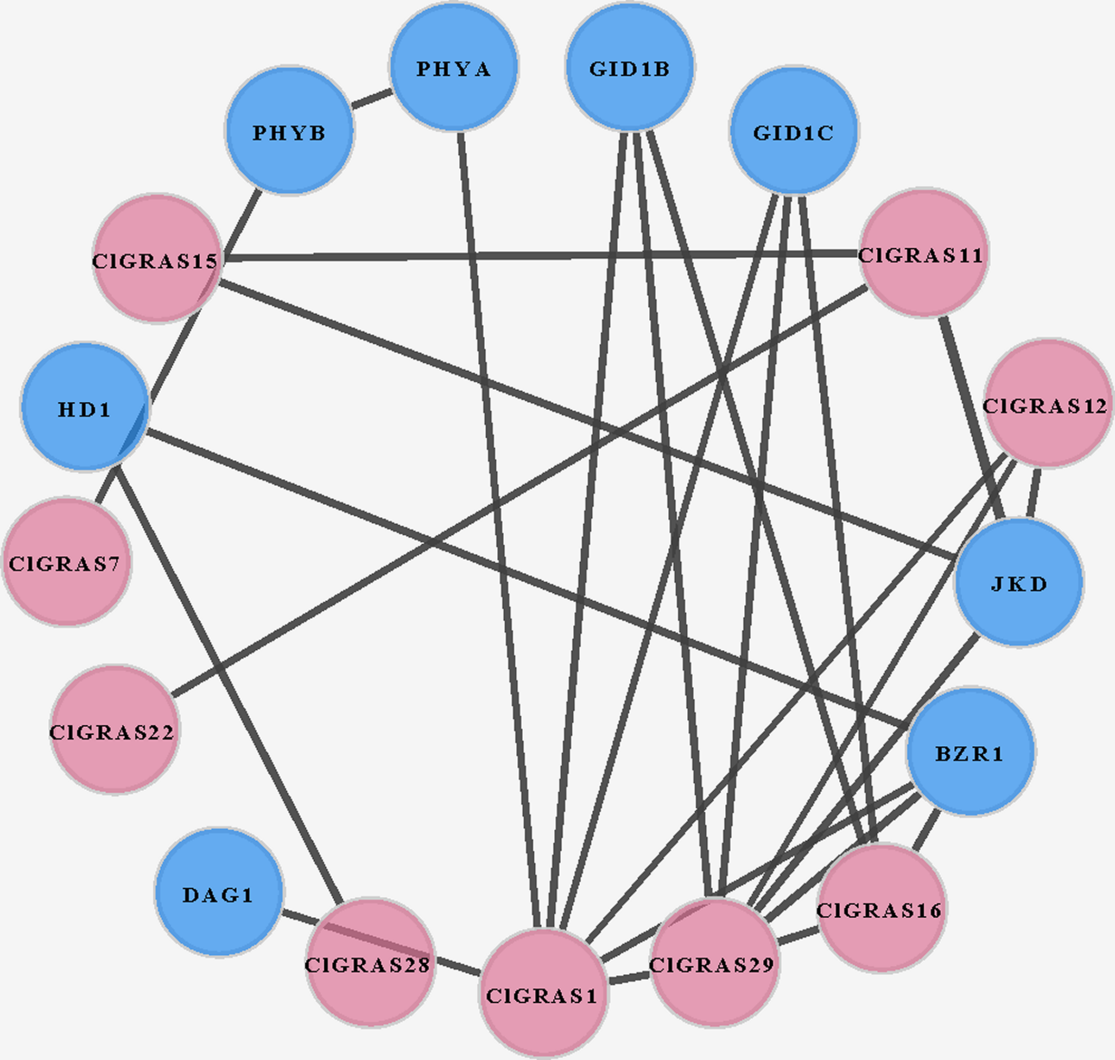
A protein-protein interaction (PPI) networks of watermelon GRAS genes. The nodes represent proteins, and the edges represent the corresponding PPI. The confidence score was required to be greater than 0.7.

To investigate the potential molecular mechanisms of watermelon ClGRAS proteins, the data only experimentally determined in the *STRING* database were selected to construct the PPI network. By using Cytoscape software, we found that a total of 25 interaction protein pairs were predicted and nine ClGRAS proteins were included in this network (Fig. 5). Apart from these ClGRAS proteins, eight proteins including PHYA and PHYB (Phytochrome A and B), JKD (C2H2-like zinc finger protein), GID1B and GID1C (alpha/beta-Hydrolases superfamily protein), BZR1 (Encodes a positive regulator of the brassinosteroid signalling pathway), HD1 (Encodes a histone deacetylase) and DAG1 (Dof-type zinc finger DNA-binding family protein) were also presented in this network. Among them, ClGRAS16, JKD, and BZR1 were paired with 4 proteins, separately; while ClGRAS11, ClGRAS12, GID1B, and GID1C were associated with three proteins, and the remaining proteins interacted with other one or two proteins. Apparently, two ClGRAS proteins exhibited the most protein-protein interactions, ClGRAS1 and ClGRAS29, which revealed that these two proteins played a key role in the whole protein interactions network. Moreover, ClGRAS16, JKD, and BZR1 featured prominently in the protein-protein network. It was illustrated that the ClGRAS protein correlated with them may possess the function of a nuclear-localized putative transcription factor with three zinc finger domains (JKD) and regulating the brassinosteroid signalling pathway (BZR1). In summary, the results obtained in the present study showed various types of protein interactions of the ClGRAS proteins. Although the ClGRASs’ interaction network needs to be further verified by experiment, our results will further expedite the identification of more essential proteins and biological modules which interacted with ClGRAS proteins and provide vital theoretical evidence for the molecular mechanisms of ClGRASs. The detailed information of the proteins in this PPI network is presented in Table S3.

### Expression analysis of ClGRAS genes in fruit flesh and rind during watermelon fruit development

**Figure 6:**
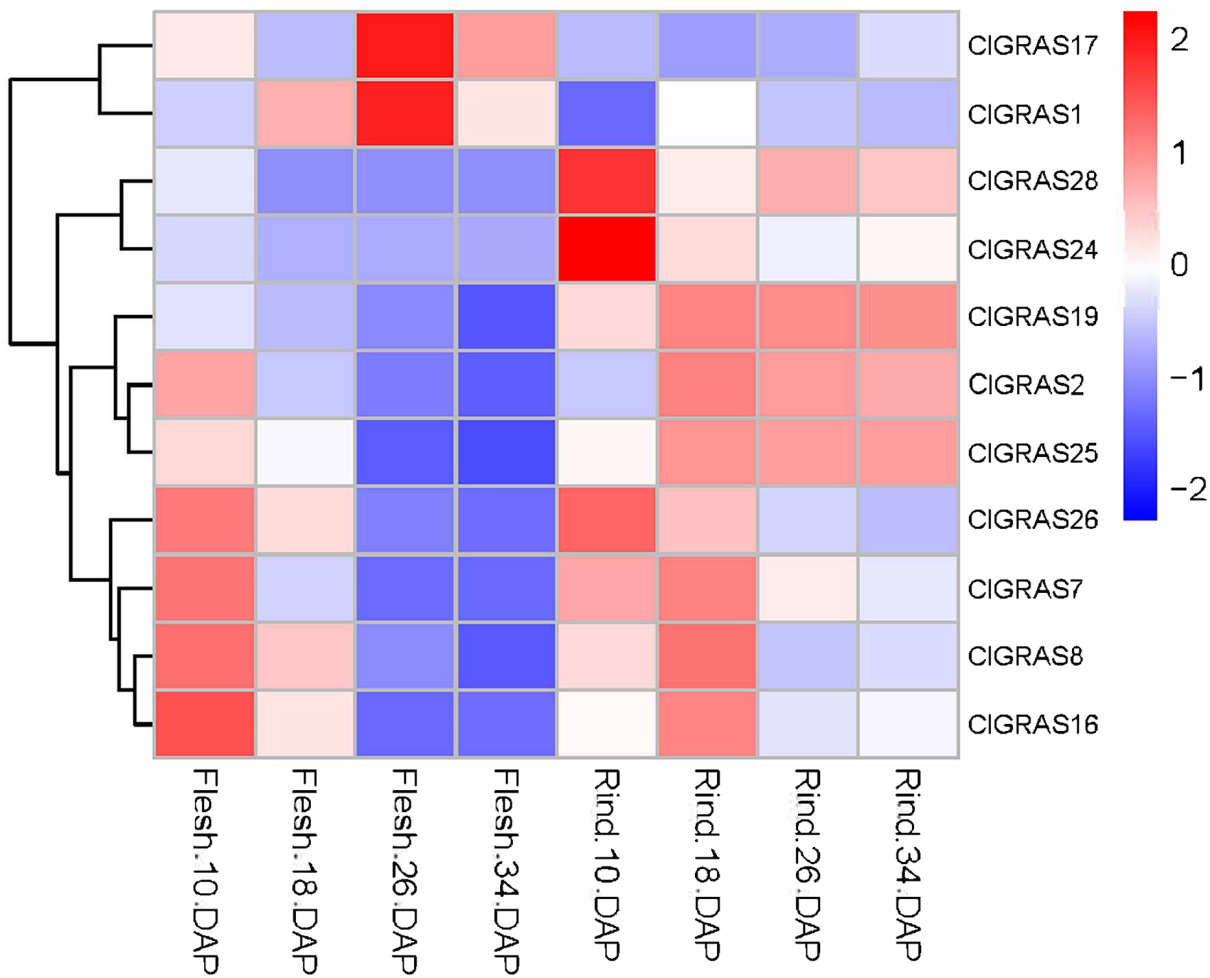
Expression profiles of ClGRAS genes in fruit flesh and rind during watermelon fruit development. Flesh & Rind_10_18, 26, 34 DAP, indicated two tissues sampled at 10, 18, 26, and 34 days after pollination.

To further evaluate the potential functions of ClGRAS genes in the growth and development of watermelon, transcriptome data derived from the fleshes and rinds of watermelon fruit at four developmental stages were applied to determine the expression levels of ClGRAS gene families. From the heat map drawn by the Rstudio (Fig. 6), a total of 26 ClGRAS genes were not expressed during the development of watermelon fruit, which might be lost their functions during evolution. Furthermore, the remaining 11 ClGRAS genes certainly participated in the regulation of fruit development and maturity. Overall, the expression patterns of these 11 genes were altered, either up-regulated or down-regulated. Of them, ClGRAS7, ClGRAS8, ClGRAS16, and ClGRAS26 had relatively high expression in the fruit flesh and rind at 10 DAP and 18 DAP, respectively, while relatively low expression levels in fruit flesh at 26 and 34 DAP. Apart from that, there existed 5 ClGRAS genes which displayed a relatively low-level expression in fruit flesh at 26 and 34 DAP. Additionally, ClGRAS1 and ClGRAS17, ClGRAS24, and ClGRAS28 were significantly expressed in fruit flesh at 26 DAP and in the rind at 10 DAP, respectively, which indicated that these genes may function in rind and fruit flesh development of watermelon. According to Gene Ontology, these differential expression genes are annotated with the 5 to 17 GO terms, such as transcription, DNA-templated (GO: 0006351), regulation of transcription, DNA-templated (GO: 0006355), and biological_process (GO: 0008150). Surprisingly, three genes, ClGRAS2, ClGRAS19, and ClGRAS25 exhibited a similar expression in the rind at 18, 26, and 34 DAP, suggesting that these three genes may have a synergistic effect in the rind development. Also, several genes have no expression at some time points, such as ClGRAS1, which was not expressed at rind tissue at 18 DAP. Collectively, overlapping but diverse expression levels of the ClGRAS genes showed that these genes might perform a variety of crucial roles in the growth and developmental processes of watermelon.

### Expression analysis of ClGRAS genes under root-knot nematode infection

**Figure 7:**
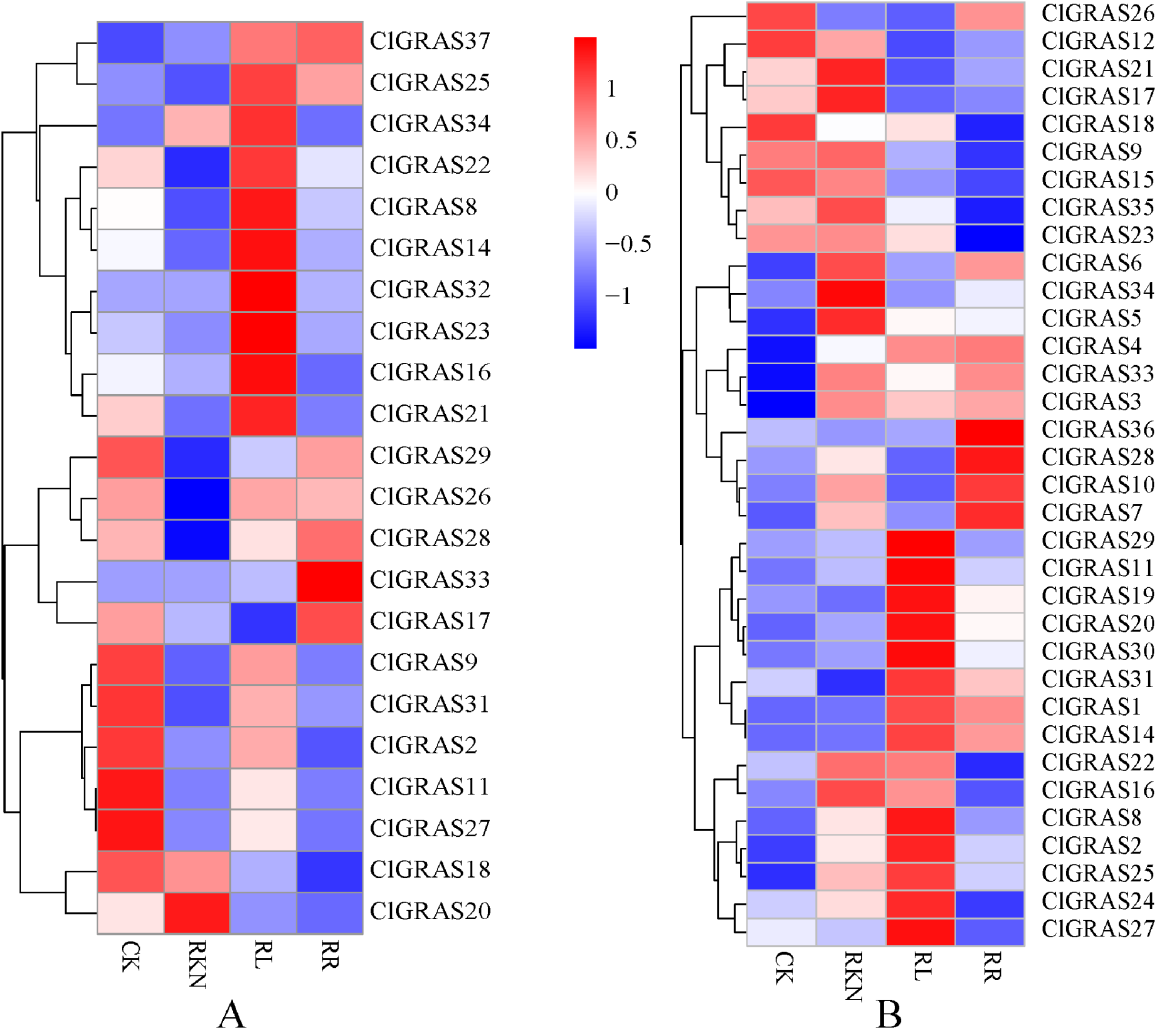
Expression patterns of ClGRAS genes in roots (A) and leaves (B). Two tissues with white light and water solution (CK), inoculation of *M. incognita* under white light (RKN), red light and water control (RL), and inoculation of *M. incognita* under red light (RR).

To gain insight into the potential functions of ClGRAS genes in response to biotic stresses, expression profiles of each ClGRAS gene in the roots and leaves of watermelon under root-knot nematode infection were investigated. As shown in Fig. 7A, under the treatments of CK, RKN, RL, and RR, expression levels of the ClGRAS genes were varied in roots. The expression of 11 ClGRAS genes (2 up-regulated and 9 down-regulated) was significantly changed by RKN treatment compared with CK. In addition, a total of 10 and 3 ClGRAS genes were respectively up-regulated and down-regulated by RL treatment compared with RKN. Moreover, compared with RL treatment, 3 and 8 ClGRAS genes were found to be (up-regulated and down-regulated, respectively) altered expression under RR treatment. Among the expression profiles of these ClGRAS genes, it was obviously found that 10 genes had high expression under RL treatment, whereas reduced in other treatment. These results illustrated that the genes played a vital role in response to red light treatment. The analogous phenomenon has existed in other genes, for instance, ClGRAS18 with RR and ClGRAS8 on RKN treatment.

We also determined the expression levels of the ClGRAS genes in leaves, and the results of changes in expression profiles are shown in Fig. 7B. A total of 8 and 3 ClGRAS genes showed apparent upregulation and downregulation under the treatment of RKN relative to CK, respectively. Additionally, there were 12 and 10 ClGRAS genes with remarkably incrementing and decreasing expression by RL treatment compared with RKN. In contrast, under the treatment of RR, only five genes had a relatively high expression, the rest of the genes were either only expressed at very low levels or not expressed. Similar to the root, there were a string of genes that were highly expressed under RL treatment, while were not expressed or only expressed at very low levels under other treatments, which revealed that these genes are putatively involved in the response to RL treatment. Similar results were also found in RKN treatment. Furthermore, based on Gene Ontology, these high expression genes are annotated with the 8 to 19 GO terms, for example, transcription, DNA-templated (GO: 0006351), transcription factor activity, sequence-specific DNA binding (GO: 0003700), cellular_component (GO: 0005575), molecular_function (GO: 0003674), sequence-specific DNA binding (GO: 0043565), regulation of transcription, DNA-templated (GO: 0006355), and biological_process (GO: 0008150).

**Figure 8:**
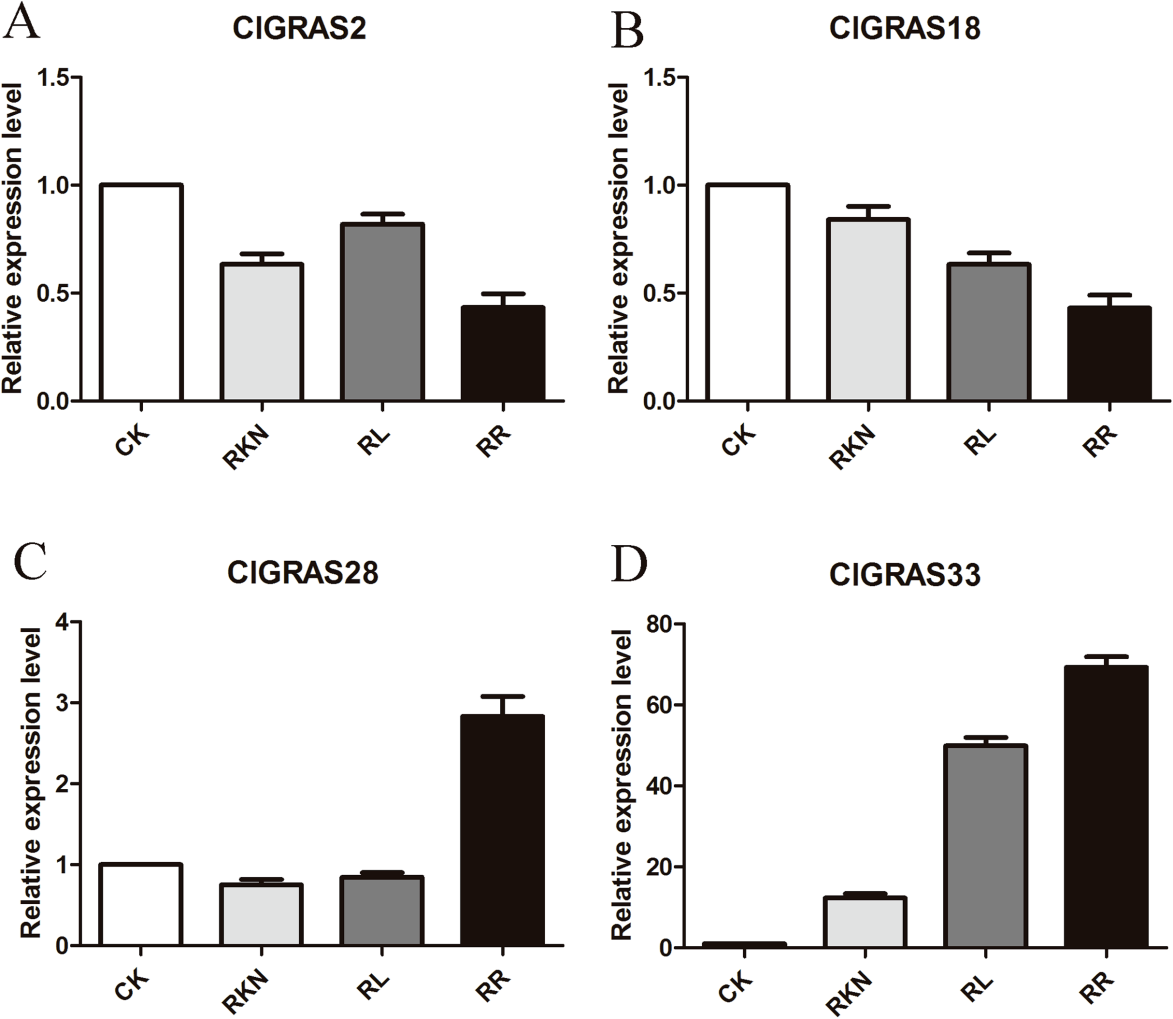
qRT-PCR results of 4 ClGRAS genes in roots. Watermelon root under the treatments with inoculation of *M. incognita* under white light (RKN), red light and water control (RL), inoculation of *M. incognita* under red light (RR) and white light and white light and water solution (CK). (A) The relative expression level ClGRAS2; (B) The relative expression level ClGRAS18; (C) The relative expression level ClGRAS28; (D) The relative expression level ClGRAS33.

**Figure 9:**
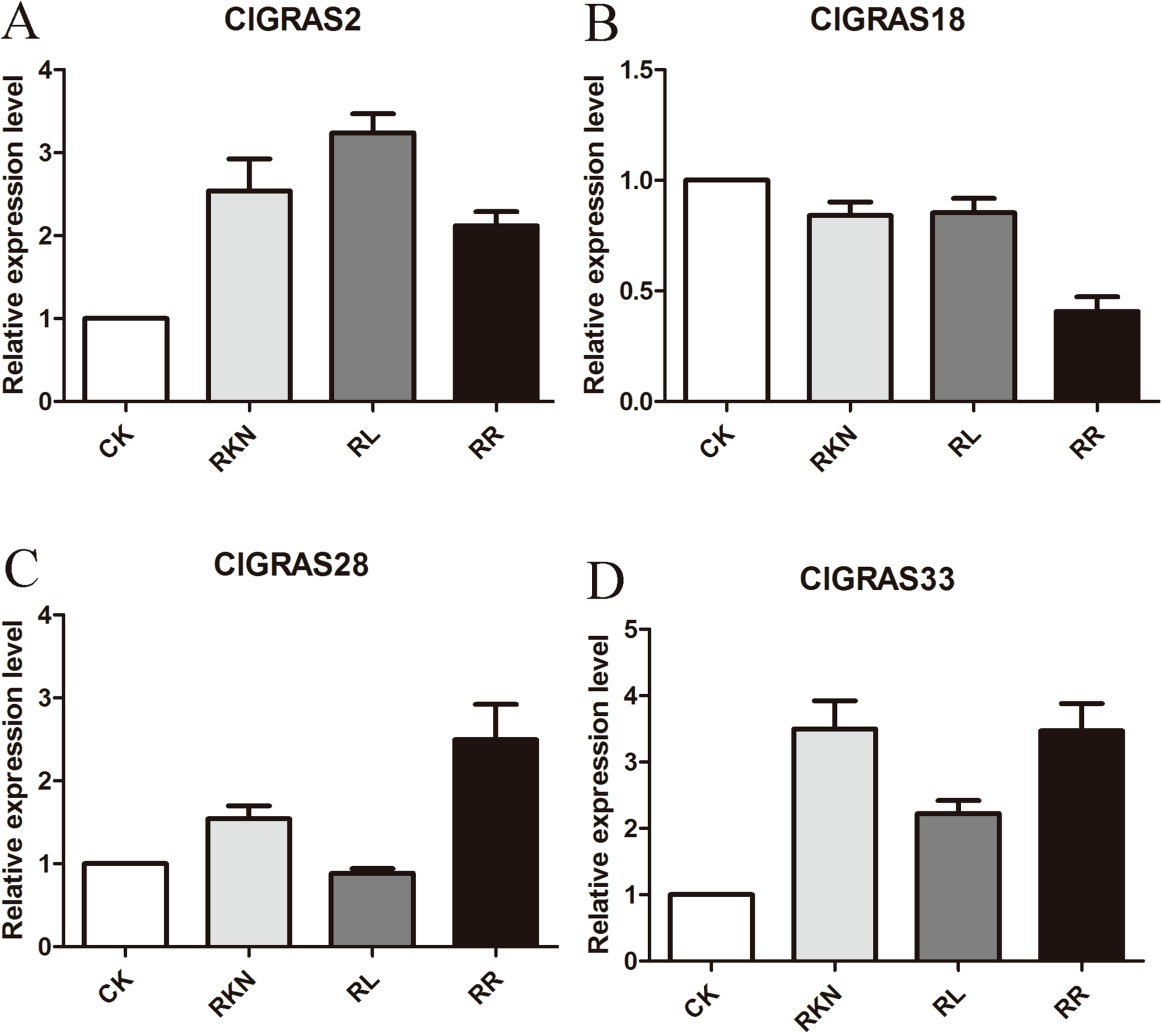
qRT-PCR results of 4 ClGRAS genes in leaves. Watermelon leave under the treatments with inoculation of *M. incognita* under white light (RKN), red light and water control (RL), inoculation of *M. incognita* under red light (RR) and white light and white light and water solution (CK). (A) The relative expression level ClGRAS2; (B) The relative expression level ClGRAS18; (C) The relative expression level ClGRAS28; (D) The relative expression level ClGRAS33.

To test the accuracy of gene expression determined from transcriptome data, we analyzed the expressions of 4 randomly selected ClGRAS genes under four different treatments by qRT-PCR (CK, RKN, RL, and RR). As showing in Fig. 8, expression levels of the 4 ClGRAS genes differed among four treatments, indicating their involvement in response to these treatments. In roots, relative expression levels of ClGRAS2 and ClGRAS18 were differentially down-regulated under another three treatments compared with CK. ClGRAS28 exhibited closely resemble expression levels by CK, RKN, and RL treatments, while showed remarkably high expression levels by RR treatment. Noticeably, the relative expression level of ClGRAS33 was extraordinarily altered by RKN, RL, and RR treatments, especially by RR treatment. Moreover, expression profiles were changed in leaves, ClGRAS2 was significantly upregulated after RKN, RL, and RR treatments, whereas down-regulated by RR treatment when compared with RKN and RL (Fig. 9). ClGRAS18 held similar expression levels by RKN and RL treatments and evidently restrained by RR treatment. It is noteworthy that ClGRAS28 and ClGRAS33 have analogous expression patterns and higher expression levels by RKN and RR treatments, however, lower in RL treatment. On the whole, the qRT-PCR results were in accord with that of previous transcriptome results.

## Discussion

Plant-specific transcription factors such as GRAS proteins are well known to be involved in photosynthesis, plant developing, signal transduction, and reproduction. Additionally, GRAS proteins have also been confirmed as homologous proteins to animal STAT proteins (Richards, Peng & Harberd, 2000), which primarily responsible for cell differentiation, reprogramming, and regeneration (Liang et al., 2012; Trenerry, Gatta & Cameron-Smith, 2011). Watermelon is an agricultural crop with high nutritional and economic values grown throughout the world. In the past decades, plenty of GRAS genes have been identified and functionally annotated in various plants. Nevertheless, there is still no comprehensive characterization and analysis of the GRAS genes family of watermelon.

With the availability of the watermelon genome and rapid development of bioinformatics analysis, herein, we performed a genome-wide identification, characterization, and expression analysis of the watermelon ClGRAS genes family. 37 ClGRAS genes were identified in the whole watermelon genome and randomly distributed on 11 chromosomes (Table 1, Fig. 1). Apparently, the vast majority of ClGRAS genes accumulate on the upper and lower parts of chromosomes, which is in accordance with other plants, for instance, in potato and tomato, suggesting conversed locations of GRAS genes during the evolution of these plants (Huang et al., 2015; Wang et al., 2019). Throughout the phylogenetic tree of watermelon, rice, and *Arabidopsis*, 10 subfamilies harbored distinct GRAS genes of 3 species (Fig. 2). It is plain to see OsGRAS and ClGRAS genes present on all subfamilies, a similar result was also observed on AtGRAS and ClGRAS genes with the exception of the subfamily Os4. These results indicated that GRAS genes were held an analogous evolution trajectory. Specifically, there are 6 and 3 ClGRAS genes contained in SHR and SCR subfamily, respectively. SHR and SCR transcription regulators were previously confirmed that they can control specification of the stem cell niche and radial patterning by radial signaling in the *Arabidopsis* root and shoot (Cui et al., 2007; Sabatini et al., 2003). Consequently, we predict these ClGRAS genes possess identical functions on root and shoot development. Moreover, it was reported that SHR protein generally interrelates with SCR and SCL23 proteins thus manipulating bundle sheath cell-fate specification (Cui et al., 2014). ClGRAS17 and ClGRAS12 genes clustered with AtSCL3 into the SCL3 subfamily. Therefore, we speculate these 2 genes and AtSCL3 may possess analogous functional role, the latter are positive regulator to integrate and maintain a functional GA pathway, integrator of GA/DELLA signaling, and the SCR/SHR pathway in root cell elongation (Heo et al., 2011; Zhang et al., 2011). Eight ClGRAS genes are members of the HAM subfamily, which is top of all subfamily, 5 and 6 GRAS genes from *Arabidopsis* and rice are also identified in this family. *Arabidopsis* HAM1, HAM2, and HAM3 were described along with a fourth, more distantly related homolog HAM4 (AtSCL15) genes functional redundancy, which modulated stem and root tips, and bud meristems (Engstrom et al., 2011). Besides, there was a study described that HAM in *Petunia hybrida* was pivotal and specifically for maintaining the shoot apical meristem and keeping stem cell maintenance (Stuurman, Jaggi & Kuhlemeier, 2002). Only one ClGRAS gene is classified as DLT subfamily members (ClGRAS33), AtSCL28, OsGRAS29, and OsGRAS19 are also encompassed in this subfamily. Of them, OsGRAS29 was found to be associated with negative feedback regulation of brassinosteroid (BR) biosynthesis, which revealed homologous gene ClGRAS33 may possess a similar function (Tong et al., 2009). Noticeably, the Os4 subfamily comprised four members: ClGRAS21, ClGRAS37, OsGRAS4, and OsGRAS43, which did not contain any *Arabidopsis* genes, showing lineage-specific gene loss in *Arabidopsis* and maybe a rice-specific subfamily. The subfamily DELLA harbored 3, 4, and 5 GRAS genes from rice, watermelon, and *Arabidopsis*, respectively. The reason for the name of this subfamily is that the presence of conserved della domain in the N-terminal zone. In the previous research, AtGAI, AtRGA, AtRGL1, AtRGL2, and AtRGL3 were confirmed to play an essential role in GA biosynthesis and negatively modulate GA signaling pathways (Piskurewicz & Lopez-Molina, 2009; Silverstone, Ciampaglio & Sun, 1998). Additionally, Tyler et al demonstrated that RGL2 is the major repressor in seed germination; AtRGA, AtRGL1, and AtRGL2 are all responsible for mediating floral development (Tyler et al., 2004). In summary, the function of GRAS genes varies between distinct subfamilies, which suggested the potential roles of ClGRAS genes played in watermelon.

Accumulating research proved that introns and exons are significant in the evolution of gene families, and the exon-intron structure analysis will assist further explore gene functions. According to the exon-intron organization of watermelon ClGRAS genes, more than 75% of genes lacked introns (28 out of 37, Fig. 3). This percentage is higher than Populus (54.7%), rice (55%), orchardgrass (56.52%), *Medicago truncatula* (64.7%), and *Arabidopsis* (67%), similar to tomato (77.4%), while smaller than *Zea Mays L* (80.23%), *Prunus mume* (82.2%), grapevine (88.46%) and potato (90%) (Grimplet et al., 2016; Guo et al., 2017; Huang et al., 2015; Liu & Widmer, 2014; Lu et al., 2015; Song et al., 2017; Wang et al., 2019; Xu et al., 2020). Variety percentage among these species revealing that the structure of GRAS genes might be a species-specific and high rate of intronless genes in the plant GRAS gene family suggesting the close evolutionary relationship of GRAS proteins. Besides, there was several genes family which also harbored plenty of intronless genes, for instance, small auxin-up RNAs (SAUR) gene family, large gene families such as pentatricopeptide repeat (PPR) containing proteins, DEAD box RNA helicases, and F-box transcription factor gene family (Aubourg, Kreis & Lecharny, 1999; Jain et al., 2007; Jain, Tyagi & Khurana, 2006; Lurin et al., 2004). Normally, many genes that lack intron are archetypical in the prokaryotic genomes. Zou et al. reported three possible reasons for the formation of numerous intronless genes in eukaryotic genomes: horizontal gene transfer from ancient prokaryotes, duplication of existing intronless genes, and retroposition of intron-containing genes (Zou, Guo & He, 2011). Subsequently, Zhang et al. proposed plant GRAS genes deriving from prokaryotic genomes of bacteria via horizontal gene transfer, which may provide a reasonable explanation for the abundance of intronless genes within the plant GRAS gene family (Zhang, Iyer & Aravind, 2012). In the present study, ClGRAS genes that possess introns were predominantly focus on the SHR subfamily, and an analogous phenomenon was observed in cotton and moss, indicating these homologous GRAS genes might perform similar functions (Zhang et al., 2018).

The specificity of gene expression patterns in plant developmental stages can supply crucial clues in regard to the possible roles of genes. Considerable evidence showed that GRAS genes are associated with plant developmental processes, for example, male gametogenesis, root tip regeneration, cell maintenance, and proliferation. The putative ortholog of the *Arabidopsis* SHORT-ROOT gene, PrSCL1, and PrSHR, members of *Pinus radiata* GRAS genes family was demonstrated to participate in the adventitious root formation in rooting-competent cuttings (Sanchez et al., 2007; Sole et al., 2008). *Castanea sativa* SCARECROWLIKE (CsSCL1) gene, dominantly expressed in roots and root primordia, is also associated with the early stage adventitious rooting (Vielba et al., 2011). Moreover, two tomato GRAS genes, SlGRAS18 and SlGRAS38, mainly expressed in breakers and red ripening stage fruits, were identified as target genes of RIN which is one of the critical earliest-acting ripening regulators (Fujisawa et al., 2013; Fujisawa et al., 2012). In the current study, the differential expression patterns of ClGRAS genes at distinct growth stages indicated their divergent functions. ClGRAS1 and ClGRAS17 significantly expressed in fruit flesh in the rind at 26 DAP and moderately expressed at 34 DAP, while low at 10 and 18 DAP, which indicated these two ClGRAS genes quite possible to modulate the later stages of flesh’s growth and development. Besides, ClGRAS1 and ClGRAS17 had relatively low expression levels in the rind 4 stages, which may reveal that both of them were flesh-specific genes and further studies are needed to determine this speculation. Interestingly, contrary to these two genes, several genes such as ClGRAS8 and ClGRAS26 had a dramatic expression difference between the early and late stages of flesh development, suggesting these ClGRAS genes predominantly involved in the early stage of fruit flesh growth of watermelon. Similar spatio-temporal expression patterns were also found in tomato, sharply increase or decrease upon pollination/fertilization either or both in stamen and ovary (i.e., SlGRAS8 and SlGRAS27), indicating their potential functions in the period of the ovary and anther developmental stages (Huang et al., 2015). In addition, another characteristic of the different plant’s GRAS genes is that members of several subfamilies harbored tissue-specific expression. Cucumber GRAS genes like members of the HAM subfamily were mainly expressed in reproductive organs, whereas members of the SCL3, HAM, and PAT1 subfamilies were highly expressed in vegetative organs (Li et al., 2020). Similarly, soybean GRAS genes in the PAT1, HAM, LISCL, SHR, SCL3 subfamily showed relatively high expression levels in the root (superior to other tissues) and nodule (Wang et al., 2020a). Besides, previous studies have documented that orchardgrass GRAS genes in the PAT1 subfamily (DgGRAS5, DgGRAS8, and DgGRAS17) and most of Tartary buckwheat GRAS genes were also highly expressed in roots (Liu et al., 2019; Xu et al., 2020).

Red light activates plant defenses more effectively compared with other monochromatic light. For instance, increased defense capability of cucumber plants to powdery mildew and enhanced systemic resistance of tomato plants against *Pseudomonas syringae* pv. *tomato* DC3000 and against root-knot nematodes in the roots after red light treatment (Wang et al., 2010; Yang et al., 2014; Yang et al., 2015). Moreover, watermelon leaves exposed to RL can enhance its systemic defense to root-knot nematodes, which are modulated by coordinated regulation of jasmonic acid- and salicylic acid-dependent signaling, antioxidant activity, and redox homeostasis was also reported (Yang et al., 2018). Previous research has shown that GRAS protein SCL13 functions as a positive regulatory component of the RL signaling pathway, mainly dependent on phytochrome B, but can also mediate phytochrome A responses (Torres-Galea et al., 2006). Herein, we perform an analysis with regard to the expression patterns of each ClGRAS gene in the roots and leaves under RL treatment and root-knot nematode infection. Ten and thirteen ClGRAS genes exhibited high expression levels in roots and leaves, respectively; however, only 2 and 6 genes exhibited relatively low expression in both the tissues (Figs. 7A & 7B). Of them, 3 and 1 ClGRAS genes being both up-regulated and down-regulated in roots and leaves, separately, indicating RL treatment can modulate the expression of several ClGRAS genes in watermelon. Interestingly, ClGRAS20 showed a contrasting expression level in the leaves and roots (noticeably up-regulated and down-regulated, respectively) after RL treatment, and ClGRAS21 was just the opposite, elucidating these two genes might be correlated with certain light-dependent biological processes in distinct tissues. Additionally, compared with RKN, the expression profile of ClGRAS genes varied under RR treatment, 7 and 6 ClGRAS genes were remarkably upregulated both in roots and leaves, while 3 and 12 genes were significantly downregulated in the two tissues (Figs. 7A & 7B). Intriguingly, the expression levels of several watermelon GRAS genes in the PAT1, SHR, and SCR subfamilies were significantly up-regulated under the treatment of RKN. This phenomenon was also found in soybean root GRAS genes; most GmGRAS genes in the PAT1 subfamily were at relatively high expression levels under saline and dehydration stresses while GmGRAS genes in the SCL3 subfamilies displayed distinct correlation patterns (Wang et al., 2020a). What’s more, functional identification of GmGRAS37 in the PAT1 subfamily indicated that GmGRAS37 can enhance drought and salt tolerance in transgenic plants by activating the expression of genes involved in abiotic stress responses (Wang et al., 2020b). Recent research reported cucumber GRAS genes in the five subfamilies were diversely expressed during low and high temperature, salinity, and exogenous phytohormone treatment. Of them, several CsGRAS genes in the PAT1, SCL3, and LISCL subfamilies have high expression levels, but low in other genes, this result revealed that these subfamilies are closely correlated with abiotic stresses (Li et al., 2020). Aside from this, GRAS genes are also associated with biotic stresses. Previously, structural and functional analysis of the grapevine GRAS genes indicates its possible divergent role in the control of stress responses. Among of them, grapevine GRAS genes in the PAT1 and SCL3 subfamilies, VviPAT4 and VviSCL3b were up-regulated upon *Botrytis cinerea* infection; VviSHR4 and VviHAM3 responded positively to Bois Noir attack, whereas some genes responded negatively (Grimplet et al., 2016). Quite of few GRAS genes in the PAT1 or other subfamilies in different plants while consistently showed high expression during abiotic and biotic stresses. This phenomenon indicated that these orthologs might share common regulatory mechanisms or pathways. In summaries, different GRAS gene members in distinct subfamilies were extensively correlated and variously expressed under environmental stresses. Moreover, the qRT-PCR results of four unevenly selected genes were consistent with that of the transcriptome. The most notable genes are ClGRAS28 and ClGRAS33; both of them are significantly upregulated under RR treatment. The former belonged to the PAT1 subfamily and main biological functions of this subfamily including being a positive regulator of phyB-dependent red light signalling, hypocotyl elongation, PhyA-specific signalling, and transcriptional regulators in the early stages of plant defence signalling (Sun, Jones & Rikkerink, 2012). The latter contained in the DLT subfamily which involved in BR signalling, negatively regulated by either exogenous or endogenous BRs; modulating BR responses, and participating in the control of rice tillering (Sun, Jones & Rikkerink, 2012; Tong et al., 2009). Besides, the homologous gene DLT negatively regulates the grain size of rice via regulating the number of cells in the glume and affecting the development of palea and lemma was also previously reported (Sun et al., 2013). Therefore, these two GRAS genes and several genes with dynamic expression patterns may be responsible for watermelon’s RL-induced systemic resistance against root-knot nematode infection and the size of watermelon. Despite this, the function of these genes may be potential targets for gene editing still needs to be determined in further research.

## Conclusions

In the current study, we performed a genome‑wide identification and characterization of the GRAS gene family by using a systematic analysis and identification of phylogenetic relationships, protein and gene structures, GO annotation, PPI networks, and tissue expression patterns during watermelon fruit development. Furthermore, we also determined the expression levels of watermelon ClGRAS genes under RL treatment and RKN infection. Transcriptome analysis and qRT-PCR experiments revealed that several ClGRAS genes might play vital roles in protecting watermelon from nematode infection. Based on the current findings, this study provides new insights into the specific role of ClGRAS genes under abiotic and biotic stress and will facilitate the exploitation of a single ClGRAS gene function.

## Supplemental Information

10.7717/peerj.11526/supp-1 Supplemental Information 1 The LOGO of watermelon GRAS protein motifs.

10.7717/peerj.11526/supp-2 Supplemental Information 2 Specific primers of watermelon GRAS genes used for qRT-PCR.

10.7717/peerj.11526/supp-3 Supplemental Information 3 The GO number of the ClGRAS protein.

10.7717/peerj.11526/supp-4 Supplemental Information 4 The detailed information of the proteins in PPI networks.

10.7717/peerj.11526/supp-5 Supplemental Information 5 The raw data of qRT-pcr results of the expression profiles of four ClGRAS genes on roots and leaves under four treatments.

## Additional Information and Declarations

### Data Availability

The following information was supplied regarding data availability: Raw data are available as Supplemental Files.
