# Supplementary figures and images for: The GRAS gene family in watermelons: identification, characterization and expression analysis of different tissues and root-knot nematode infestations
Source: PeerJ. 2021 May 26;9:e11526. doi: 10.7717/peerj.11526 (PMC8164414; doi:10.7717/peerj.11526)

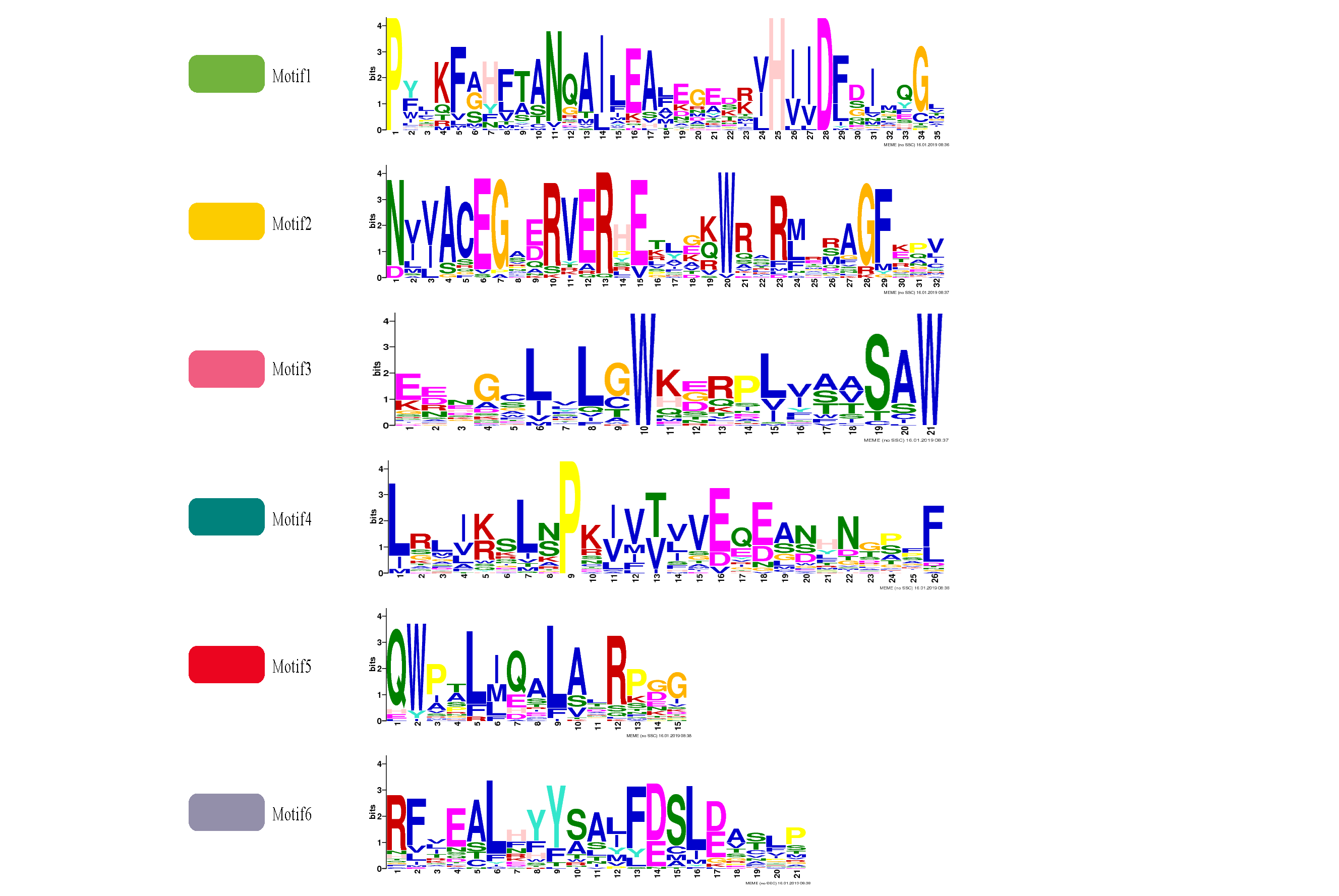

Supplement: Supplemental Information 1 [file peerj-09-11526-s001.png]
