# Supplementary material for: The GRAS gene family in watermelons: identification, characterization and expression analysis of different tissues and root-knot nematode infestations
Source: PeerJ. 2021 May 26;9:e11526. doi: 10.7717/peerj.11526 (PMC8164414; doi:10.7717/peerj.11526)
Supplement: Supplemental Information 2 [file peerj-09-11526-s002.docx]

**Table S1:** **Specific primers of watermelon GRAS genes used for qRT-PCR.**

| **GRAS genes** | **Specific primers** |
| --- | --- |
| ClGRAS2 | GATTTCGAGCGGTAAGGTTAGG  CTCAACCTCGGACCTTCATTT |
| ClGRAS18 | CTTTGCTCAAGCGGTACAAAC  CCACCACCTTCTTCTTCTTCTC |
| ClGRAS28 | TTCCGAGATCGAGGCAAATTAT  TCGAAGTACGATGCGGTAAAG |
| ClGRAS33 | TCTGTCACGGAACCAGTTAATC  GCACAAGCACAAAGCATCTC |
